# Perception of medical students and residents about virtual interviews for residency applications in the United States

**DOI:** 10.1371/journal.pone.0238239

**Published:** 2020-08-31

**Authors:** Ali Seifi, Alireza Mirahmadizadeh, Vahid Eslami

**Affiliations:** 1 University of Texas Health at San Antonio, San Antonio, Texas, United States of America; 2 Department of Epidemiology, School of Health, Shiraz University of Medical Sciences, Shiraz, Iran; 3 Department of Neurology, University of Texas Health at San Antonio, San Antonio, Texas, United States of America; Imam Abdulrahman Bin Faisal University, SAUDI ARABIA

## Abstract

**Introduction:**

Residency applications via virtual-interview could potentially mitigate the extensive cost and time required for customary in-person interviews. We outline the perception of medical students and residents on the use of virtual-interview for residency applications in lieu of in-person interviews.

**Methods:**

We obtained 1824 responses from medical students and residents through an online questionnaire between March2019-Feb2020 in Texas-United States. The survey had 11 statements (five in favor of in-person interviews and 6 in favor of virtual interviews) that respondents could rank on a 5-point Likert scale. All statements’ scores were summed based on the response given by each participant to create a total score between 11 and 55. The perception of the two groups was analyzed using an independent sample T-test and ANOVA.

**Results:**

We received a total of 1711 responses from medical students and 113 from medical residents. Respondents were more female (82.2% of medical students and 47.8% of residents), with a mean age of 22.87±3.42 years old for medical students and 28.72±4.35 years old for residents. Both groups preferred in-person interviews; however, the residents were significantly more in favor (P = 0.03). Both groups agree that virtual-interviews should be as an option, though this was considerably higher in the medical students (P = 0.001). In the multivariate analysis, “travel distance” and “type of medical school” had a significant impact on choosing the virtual-interviews in both groups (p<0.01).

**Conclusions:**

In-person interviews are favored by both medical students and residents compared to virtual-interview services in normal circumstances. However, both groups agree that programs should offer the option of having virtual-interviews as an available choice. Distance to an interview location and the type of medical school were the factors that had a significant impact on perception of using virtual-interviews. Knowing about the applicants’ attitude toward residency interviews and the national circumstances are essential when preparing the interview guides. Our findings are limited by the small sample size and the low response rate. Further extensive studies are warranted to better understand the perception of residency applicants toward virtual-interviews to improve the interview process in the United States.

## Introduction

Every year in the United States, medical students continue to confront the increasingly large financial burden and complex time management necessary for travel during residency interview season. The Association of American Medical Colleges (ACGME) has changed the in-person interviews to a virtual format for 2020–2021 residency interviews [1]. Given the competitive nature of residency interviews, online meetings can be concerning for applicants. Every year in the United States, medical students continue to confront the increasingly large financial burden and complex time management necessary for travel during the residency interview season. Applying to residency in the United States is a complex and cumbersome process, both emotionally and financially. It is a lengthy process that starts with selecting a specialty during the medical school and sending the application to the desired institutions in September. The programs review the applications and offer an in-person interview if the applicant meets the qualifications. Tens of travels will follow this to each institution for the interview. Eventually, the applicants and programs rank each other, and the future residents will be selected, if the applicant will be matched, in March of each year [2]. According to the Association of American Medical Colleges (AAMC), the average number of residency applications per student in U.S. public medical schools was 60.5 in 2018, up from 48.8 in 2014 [3]. A study published in 2014 found that the average number of interviews for fourth-year medical students was 12.3, and 65.7% of students spent between $1,000-$5,000 during the interview season [4]. Expenses include travel (airfare, lodging, transportation, etc.), meals, clothing, application fees, match fees, and other additional costs. As the average number of applications per student in the U.S. continues to rise, so might the average cost of total expenses for a residency interview season. In 2015, the AAMC found that 79% of fourth-year medical students thought the expenses related to travel were overly burdensome [5]. Time management also lends its own challenges, as one study found the average applicant commits 20 days away from medical education to pursue their residency interviews [6]. More so now than ever are researchers looking for ways to alleviate this growing strain on an already overloaded population of limited financial means. Recently, studies have found the use of web-based interviews as a possible solution, as they are cost-effective, reduce scheduling conflicts, and decrease time away from educational pursuits [7].

Although this may prove to be a valuable solution, in theory, it requires the approval, and thus, participation of medical students and faculty. Our study aims to evaluate the current perception of medical students and residents on the use of virtual-interviews for residency applications in the United States in lieu of in-person interviews.

## Methods

This is a cross-sectional study that was done via an electronic questionnaire in Texas, United States. The invitation link was active March 2019-Feb 2020. Two questionnaires, one designed specifically for medical students and one for residents, were made to collect our data. Content and face validity of the questionnaire were corrected and approved by two experts of virtual learning and by one epidemiologist. The questionnaire for medical students asked prospective questions regarding utilizing virtual-interviews for residency (i.e., how much they think they will spend or how they feel it will affect their interviews), whereas the residency questionnaire asked retrospective questions (i.e., how much did they spend or how did it affect their interviews). Both questionnaires started by collecting basic demographic information, including age, gender, ethnicity, and medical school location. Then, respondents were asked about the minimum distance in which they may or did consider doing an interview over virtual system, as well as the dollar amount they anticipate spending or did spend during the interview season. Afterward, there were eleven questions that respondents could rank on a scale from 1–5 regarding how much they agreed with the question statement, one being completely disagree and five being completely agreed on a 5-point Likert scale. The eleven questions were constant between both questionnaires. Six question statements were in favor of virtual-interviews, and five-question statements were in favor of in-person interviews. In order to standardize the ranking of the answers, the score of the statements in favor of in-person interviews were reversed for the analysis. Therefore, score of five always meant in favor of virtual-interviews for the final univariate and multivariate analysis. In a univariate analysis using an independent sample T-test or ANOVA, all eleven questions were summed by their 1–5 ranking answer responses given by each participant to create a total score between 11 (respondent answered 1 to all 11 question statements) and 55 (respondent answered 5 to all 11 question statements), with a score of 33 being the neutral average of all perceptions. At the end of the questionnaire, participants were able to add additional free text comments on this topic.

Links to the questionnaires were sent by email to the medical schools in Texas and residency programs at the University of Texas Health at San Antonio. The programs had the freedom to send the questionnaire to the audiences or not, based on their policies. Institutions were also allowed to give the link of the survey to their residency applicants (American or foreign medical school graduates) and the international physicians or students who were rotating, shadowing, researching, or working in those institutions who were applying into a residency in the United States.

To estimate an “approximate” response rate, we calculated each group's response rate by dividing the number of responses in each group to the total population of medical students in Texas (6,700) and the residents in our home institution (798). Participation was totally voluntary, anonymous, and free. There was no payment, reward or gift provided to participants.

Gender, race, location of medical school, cost, and distance of travel were included in the univariate model. In multivariate analysis, factors that were P<0.2 in univariate analysis were included in a multivariate model. A P-value of less than 0.05 was considered significant in all of the analyses.

The Institutional Review Board (IRBs) of the University of Texas Health at San Antonio approved this study under IRB number HSC20190163E. Statistical Software SPSS for Windows (version 21.0; SPSS/IBM, Chicago, IL) was used to analyze the data.

## Results

We received a total of 1711 responses from medical students and 113 from medical residents. The approximate response rate of medical students was 25.54%, and 14.16% for the residents. The student group was mainly female (82.2%), of white ethnicity (53.5%), and from an American medical school (65%) with a mean age of 22.87±3.42 years old. The resident group was 47.8% female, mainly of white ethnicity (93.8%), and graduated from an American medical school (63.7%) with a mean age of 28.72±4.35 years old (Table 1). 15% of the residents had previous experience of the virtual-interviews for their residency application and traveled on average up to 2,737.98 miles and spent, on average, $4995.23 during the interview season. When asked about the minimum distance in which an applicant might start to consider virtual-interviews rather than in-person, medical students leaned towards longer distances while residents varied greatly. When medical students were asked to estimate the average dollar amount an applicant typically spends on the interview season, most medical students estimated $1000–5000 (34.2%), with residents concurring with that value, reporting to have spent $1000–5000 (50%) during their interview season (Table 1).

**Table 1:** Demographic characteristics of study participants.

Variables		Student		Resident	
**Age**	• Mean ± SD	22.87±3.42		28.72±4.35	
	• Median	23		29	
	• Range	30 (25–45)		31 (22–43)	
		**No.**	**%**	**No.**	**%**
**Sex**	• Female	1406	82.2	54	47.8
	• Male	297	17.4	56	49.6
	• Other	8	0.4	3	2.7
**Race**	• White	916	53.5	106	93.8
	• Asian	408	23.8	-	-
	• Hispanic, Latino, or Spanish origin	187	10.9	-	-
	• Black	142	8.3	7	6.2
	• Native Hawaiian or other Specific Islander	26	1.5	-	-
	• American Indian or Alaskan Native	24	1.4	-	-
	• Other	8	0.5	-	-
		0	0	0	0
**Medical School**	• American Medical School	1113	65	72	63.7
	• Foreign Medical School	598	35	41	36.3
**At what distance would you begin to consider having your residency interview done over Virtual communication? (miles)**	• Same city	52	3.0	2	1.8
	• 300 miles (LA to Las Vegas)	171	10.0	12	10.6
	• 1000 miles (LA to Denver)	279	16.3	26	23
	• 1500 miles (LA to Houston)	183	10.7	12	10.6
	• 2500 miles (LA to NYC)	326	19.1	26	23
	• Different country	700	40.9	13	11.5
**Approximately, how much do you think you will spend/or spent during your residency interview season? ($)**	• < $1,000	215	12.6	17	20.7
	• 1000 - < $5,000	585	34.2	41	50
	• 5000 - <10,000	448	26.2	18	22
	• 10,000 - <15,000	249	14.6	5	6.1
	• 15,000 - <20,000	103	6.0	0	0
	• 20,000 - <25,000	55	3.2	1	1.2
	• ≥25,000	56	3.3	0	0
**Total**		**1711**	**100**	**113**	**100**

### Perception on the virtual-interviews versus in-person interviews

Overall most of the statements were scored in favor of in-person interviews (9 out of 11 statements in both group), aside from a few exceptions (Table 2). The mean score for the medical students who demonstrated worrisome about the time required away from medical school for residency interviews was higher than the residents (3.84±1.12 vs. 2.93±1.35, P = 0.001). Both groups showed unease regarding travel expenses; however, this concern reached a higher mean score by the medical students (4.13±1.04 vs. 3.83±1.21, P = 0.01). Medical students also had a higher mean score regarding experiencing technical difficulties during online virtual-interviews compared to their graduate counterparts (3.98±1.18 vs. 2.92±1.4, P = 0.001). At the end of our survey, both groups were asked about whether virtual-interviews should be an option for them for residency interviews, to which most participants agreed in both group, however, the medical student group reached a higher mean score (4.18±1.06 vs. 3.74±1.21, P = 0.001) (Table 2). The two out of eleven statements that gained a higher mean score toward virtual-interviews in both groups were;” I think an interview over virtual communication is less stressful than an interview in person” and “I think programs should offer both options of interviewing in-person and over virtual communication,” (Table 2).

**Table 2:** Mean agreement score for statements regarding virtual interviews vs. in-person interviews.

No.	Statement Questions	Mean (1–5 ±SD)		
		Student *(n = 1711)*	Resident *(n = 113)*	*P*
1	It is important that you visit the location (building, city, etc.) of the residency program. *	**4.36±0.9**	**4.29±1.1**	**0.55**
2	Having a residency interview over virtual communication would make you seem as much of a serious or dedicated applicant as someone who went to their interview in person.	**2.86±1.26**	**2.51±1.32**	**0.005**
3	It is really important for you to meet the residents and staff of your residency program. *	**4.44±0.83**	**4.31±1.02**	**0.18**
4	I think having a residency interview over virtual communication would affect the personal connection to your interviewer compared to someone who went in person. *	**3.91±1.12**	**4.04±1.15**	**0.26**
5	I think an interview over virtual communication is less stressful than an interview in person.	**3.47±1.26**	**3.31±1.26**	**0.18**
6	I am concerned about / will have difficulty affording all the travel needed for residency interviews.	**4.13±1.04**	**3.83±1.21**	**0.01**
7	I am concerned about the time that I have to be away from school during the in-person residency interviews.	**3.84±1.12**	**2.93±1.35**	**0.001**
8	I believe I can be in my goal residency position even by doing a virtual communication interview.	**3.40±1.14**	**3.26±1.34**	**0.26**
9	If I have to participate in a residency interview over virtual communication, I would be worried about computer technical difficulties. *	**3.98±1.18**	**2.92±1.4**	**0.001**
10	If I had an interview via virtual communication, I think it should be done in a special facility rather than from my phone or from my home computer. *	**3.53±1.31**	**3.56±1.43**	**0.03**
11	I think programs should offer both options of interviewing in-person and over virtual communication.	**4.18±1.06**	**3.74±1.21**	**0.001**

*Favorable statements for in-person residency interviews (reversed in analysis). Score of 5 is the highest agreement with the statement and score of 1 is the lowest agreement. The mean score for each question is a number between 1 and 5: for the *favorable statements 5 is the highest score in favor of in-person and for the unfavorable statements, 5 is in favor of virtual interview.

### The residency specialties

When asked which residencies are most conducive to interviews being done over virtual-interviews, medical students most often chose Surgery and Internal Medicine, while residents chose Family Medicine and Internal Medicine (Table 3). Medical students chose Physical Medicine & Rehabilitation and Urology as least suitable for virtual-interviews, while residents considered Neurosurgery and ENT unsuitable. In summary, medical students and residents considered non-surgical residences in general as most suitable for virtual-interviews.

**Table 3:** The specialties that the medical students plan to apply, and the residents already applied and their perception regarding the most appropriate specialties they can be offered by virtual interviews. Participants could select more than one specialty.

	Type of Residency	Plan to go into				Is OK to do over Virtual Interview			
No.		Student		Resident		Student		Resident	
		No.	%	No.	%	No.	%	No.	%
1	Surgery	319	18.6	26	23	728	42.5	24	21.2
2	Internal Medicine	643	37.6	19	16.8	681	39.8	51	45.1
3	Emergency Medicine	353	20.6	10	8.8	519	30.3	27	23.9
4	Pediatrics	452	31.7	11	9.7	444	25.9	43	38.1
5	OB-GYN	413	24.1	3	2.7	402	23.5	24	21.2
6	Family Medicine	813	47.5	4	3.5	335	19.6	56	49.6
7	Neurosurgery	292	17.1	17	15	306	17.9	19	16.8
8	Psychiatry	600	35.1	3	2.7	229	13.4	39	34.5
9	Anesthesiology	492	28.8	5	4.4	228	13.3	31	27.4
10	Dermatology	618	36.1	3	2.7	222	13	34	30.1
11	Ophthalmology	653	38.2	18	15.9	190	11.1	23	20.4
12	Orthopedics	334	19.5	1	0.9	182	10.6	22	19.5
13	Radiology	630	36.8	9	8	142	8.3	48	42.5
14	ENT	414	24.2	0	0	101	5.9	20	17.7
15	Urology	424	24.8	2	1.8	68	4	22	19.5
16	Physical Medicine and Rehabilitation	545	31.9	0	0	62	3.6	38	33.6
17	No opinion or other	54	3.4	4	3.5	23	1.3	18	15.9

### Evaluation of the perceptions

In a univariate analysis of the participants’ responses, gender had no significant impact on the perceptions shared related to virtual interviews in either the medical student group (P = 0.21) or the resident group (P = 0.91). Likewise, ethnicity had no significant impact either (medical students P = 0.24, residents P = 0.21) (Table 4). In a univariate analysis, the perceived or actual interview travel costs of medical students and residents had no significant impact on their perception toward virtual-interviews (P = 0.51, and P = 0.40, respectively), however, those who were planning to spend or who had already spent higher dollar amounts were generally in favor of virtual-interviews. Travel distance was a significant factor for medical students when electing for virtual-interviews. Students who considered doing virtual-interviews even in a relatively shorter distances, had a significantly higher likelihood of choosing question statements in favor of virtual-interviews (P<0.01). However, residents, when looking retrospectively, did not think travel distance should impact the decision to consider doing a virtual-interview (P = 0.39).

**Table 4:** Univariate comparison of agreement with virtual interview*.

Variables		Student		Resident	
		Mean ± S.D.	P**	Mean ± S.D.	P**
**Sex**	• Female	31.75±5.7	0.21	30.59±5.9	0.91
	• Male	31.26±6.2		30.48±5.5	
**Race**	• White	31.39±5.6	0.24	30.31±5.3	0.21
	• Asian	31.83±6.3		-	
	• Hispanic, Latino, or Spanish origin	32.44±5.7		-	
	• Black	31.82±5.9		32.85±5.2	
	• Native Hawaiian or Other Specific Islander	30.88±5.4		-	
	• American Indian or Alaskan Native	32.54±5.6		-	
	• Other	33.88±5.6		-	
**Medical School**	• American Medical School	30.5±5.7	**<0.01**	29.5±4.6	**0.009**
	• Foreign Medical School	32.27±5.8		32.17±5.9	
**Approximately, how much do you think you will spend/or spent during your residency interview season? ($)**	• < $1,000	31.46±5.5	0.51	32.23±3.5	0.40
	• 1000 - < $5,000	31.40±5.7		30.19±5.6	
	• 5000 - <10,000	32.06±5.8		29.44±3.9	
	• 10,000 - <15,000	31.20±6.0		30.4±4.9	
	• 15,000 - <20,000	31.58±5.6		35±0	
	• 20,000 - <25,000	32.18±6.4		-	
	• ≥25,000	33.71±7.4		-	
**At what distance would you begin to consider having your residency interview done over virtual communication? (miles)**	• Same city	33.02±7.4	**<0.01**	30.5±3.5	0.39
	• 300 miles (L.A. to Las Vegas)	34.70±5.8		30.25±2.5	
	• 1000 miles (L.A. to Denver)	33.07±5.3		30.11±5.1	
	• 1500 miles (L.A. to Houston)	31.55±4.8		28.9±5.5	
	• 2500 miles (L.A. to NYC)	30.37±5.1		29.1±4.9	
	• Different country	30.89±6.1		32.78±6.6	
**Total**		31.66±5.83		30.47±5.26	***0*.*03***

Total scores were between 11–55, with a score of 55 being the highest interest in virtual-interview and score 11 being the lowest interest; a score of 33 was neutral.

* Favorable statements for in-person residency interviews have been reversed.

**Independent sample T-test or ANOVA.

When combining all the scores together in both groups, although both medical students and residents leaned toward preferring in-person interviews, residents were significantly more in favor (P = 0.03) (Table 4).

In multivariate analysis with factors that were statistically significant, only “travel distance” and “type of medical school” had a significant impact when considering the virtual-interviews as a potentially beneficial alternative to the in-person residency interviews in both groups (p<0.01).

### Open ended comments

In the open-ended comment section, 44.25% of residents and 58.45% of medical students wrote a type of comment. The major theme of the free text comments was that the in-person interviews are invaluable. However, 23.38% of students and 26.55% of residents, in some way, suggested that virtual-interviews are good and can be offered as an option. They indicated somehow that virtual-interviews could be an appropriate alternative in one or some of the following conditions: the preliminary internship interviews, the screening process of residency application reviews, when there is a need for the second visit after the in-person interviews, for post-match scramble process and when there is travel difficulty. One medical student commented,” virtual interviews will help the applicants with a disability or when they have travel limitations.” And a resident wrote “virtual interviews should be offered to the one-year preliminary internships since this is not the home institution for the residency in most cases.”

## Discussion

Our study showed that although medical students and residents agree to consider virtual-interviews as an option, both groups prefer in-person residency interviews. In a world of growing technology that holds efficiency and speed in high regard, it seems a natural fit to utilize virtual technologies for the interviews that would otherwise necessitate large amounts of money and travel distance. However, the virtual interviews' efficiency must be more important than the in-person social connection between an interviewee and interviewers, the meeting with potential future staff, and the visiting of the hospital environment.

According to our survey, both residents and medical students demonstrated concern about the cost of residency interviews, with medical students being significantly more concerned. As stated in the AAMC “Cost of Applying to Residency Questionnaire Report,” the fourth-year medical students spent on average $3,422.71 per student for their interview season, including travel costs, lodging, and meals, 79% of whom agreed the cost of travel was overly burdensome [5]. This AAMC report is consistent with the data which was obtained from our survey. To make matters worse, the AAMC predicts that “the process may become more burdensome as the residency match becomes increasingly competitive with recent advising strategies focusing on application to an increasing number of programs” [5]. There is also growing pressure on medical students to perform away rotations at different institutions, with the overall cost of an away rotations per student being roughly $1,839. Collectively, all of these expenses on an already economically strained population can prove daunting. As one medical student stated in the AAMC report, the residency interview season is “very expensive,” but it doesn’t "feel like you have a choice because this is pretty much the most important and influential part of med school." Our survey highlights that 27.1% of medical students believe they will spend >$10,000 during residency interviews, while only 7.3% of residents reported spending that much. Therefore, perhaps residents saw the process as a more manageable, affordable, and/or justified in cost than previously thought. In addition, residents may have grown apathetic to the now seemingly small cost of residency interviews, especially when compared to their medical student counterparts, who have little to no income and hold excessive debt. This concern towards the cost of attending in-person interviews is most likely one of the largest factors to influence a shift toward virtual interviews. Performing an interview via virtual system would remove the cost for travel, fuel, meals, and lodging.

Medical students and residents also demonstrated concern about the time needed away from school to attend their in-person interviews. Although there is not much data in the literature regarding the average number of days needed away from school during the interview season, the average fourth-year medical student will attend 12.3 interviews [4] and one research study found that each urology resident spent on average 20 days away from school to attend their interviews [6]. Those 20 days for fourth-year medical school come during a time in which most medical students are completing sub-internships, away rotations, performing research electives, or even enjoying time off. Performing interviews via virtual system would greatly combat this ordeal, as many online interviews are 30 minutes to 1 hour long, after which medical students can resume their routine activities. Residents were significantly less concerned than medical students about the time required away from school. Perhaps the time away from school required for residency interviews is not as unmanageable or burdensome as often thought by medical students, with residents having the true hindsight to realize it was actually doable.

Medical students who began to consider virtual-interviews at relatively shorter distances to their interview locations were significantly more likely to side with virtual-interviews overall, whereas students who only wanted virtual-interviews at extreme distances were more likely to agree with question statements in favor of in-person interviews. This could suggest that distance, along with the time waste and monetary expense, is the single most important factor that virtual-interviews can help.

Based on AAMC, there are two types of virtual interviews provided. Live virtual interviews which are based on the virtual conference technology to connect applicants with the interviewer in real-time. In this method, applicants sit face-to-face with the interviewer(s) and answer his or her questions. The second type is an asynchronous or on-demand, virtual interview in which there are no interviewers present. The applicant will be asked to respond to questions presented via text or prerecorded video. The responses will be recorded by a webcam and shared with reviewers at a later time [8]. Both types of virtual-Interviews offer the advantages of being a more affordable and time-efficient alternative to in-person residency interviews for a population that is already economically disadvantaged and time-constrained. However, there are a few large factors that dissuade medical students and residents alike from the possibility of viewing virtual-interviews as a truly equal alternative. Two large factors that medical students and residents unanimously view as greatly important are visiting the location of their future residency and meeting the staff. Medical students will often be moving to a new city, state, or area of the country. The residency program that they secure will be their program, and the city they live in, for multiple numbers of years. Therefore, it can be expected that visiting the city, experiencing the surrounding culture, and examining the city's infrastructure are very important aspects when judging a residency program. In addition, actively going to the program and visiting the staff can have more positive impressions. Both medical students and residents seem to agree that if they were to perform an interview via virtual-interview, they would seem less dedicated or serious compared to an applicant who went to their interview in person.

In a study examining the effects of implementing videoconference interviews for an adult reconstruction fellowship for orthopedic surgery, 34% of videoconference interview candidates stated that the videoconference interviews had an unfavorable impact on their ranking of the program, with many subjective comments from applicants stating "on-site in-person interviews provide more information regarding the people, facility, culture, location, city, and 'feel' of the program" [9]. These benefits are not easily available via virtual-interview, and if virtual-interviews were to be implemented, applicants would have to judge residency programs without assessing the city or staff. According to our survey and previous research, however, these seem to be essential factors that medical students and residents alike are unwilling to forgo.

Performing an interview via a virtual system would require less planning, expense and time management. Perhaps demonstrating the willingness to undergo these stressors indicates a larger desire for the program. According to the previously cited study, 15% of the orthopedic residents felt that they did not have adequate opportunity to portray themselves to their satisfaction via the videoconference interview [9].

Residency interviews are an opportunity for the programs to gain important information about applicants' interpersonal and communication skills. Interviews play a vital role in helping both parties find out whether there is a good fit between the applicant and the program [10].

Generally speaking, the residency programs always try to select the applicants that best match to their institutions. However, this selection is a complex process and time-consuming. In order to improve the selection process, the trend is that programs move toward a structured interview process [8] and programs have been using various strategies. For example, some residency programs use the "Multiple Mini Interview" (MMI) formats to select their resident. MMI is an interview format consisting of six-10 interview stations, each focused on a different question or scenario [11]. However, the applicants had mixed feelings about MMI for residency interviews. A study on Emergency Medicine interviews found MMI may have a negative impact, although integration of MMI stations and traditional unstructured interviews was more positive [12]. Another residency program developed a "Fast Interview Track" (FIT) system for interviews, a less structured form of MMI. They found that although the structured MMI used in other studies may have left applicants without a sense of personality and atmosphere of the programs, FIT sessions provided a more comprehensive view of the programs [13]. In another study, researchers found that "behavioral interviewing" can provide predictive information regarding the success of residents in their program [14]. "Behavioral interviewing" established by industrial psychologists emphasize that past performance is the best indicator of future performance [14]. Another example of the programs' efforts to improve and ease the interview process is using the virtual systems. However, the comparison of standardized video interviews (SVI) to traditional in-person interviews was recently investigated by two studies, and they both found a positive correlation between SVI and in-person interview scores [15, 16]. In addition, Husain et al. investigated how SVIs affect an applicant's likelihood to be invited for an interview. They found that SVIs only decreased the likelihood of being invited to interview in 3.1% of applicants [17]. Authors believe that although we should consider virtual-interviews as an acceptable alternative to in-person residency interviews, the interviewee's interpersonal skills’ evaluation could be negatively affected. Interpersonal skills include verbal and non-verbal communication, as well as the ability to listen, engage, and show emotion [18]. This could be why medical students and residents also agree that having an in-person interview over virtual-interview would be detrimental to the personal connection to their interviewer, as those skills are more readily observable in-person and throughout their interview day.

As in-person interviews have their unique difficulties, so do virtual-interviews. Namely, our study showed that medical students are more concerned about experiencing technical difficulties during a virtual-interview than their resident counterparts. Technical difficulties are typically not experienced during in-person interviews, and are especially worrisome, as the interviewee has no control over the issue but can still have their interview negatively impacted. According to our survey, 15% of residents underwent a virtual-interview for their residency, which could be why they are more trusting of virtual-interviews or at least aware that interviewees can perform test calls the week before a planned interview.

Medical students and residents suggested that if virtual-interviews were to be implemented, it might be better to be performed at special facilities rather than from a mobile device or home computer. This could definitely lower unease towards experiencing technical difficulties, but this would once again introduce the initial problems of travel and expense. Applicants need to know how to use basic technology for the interview. They need a suitable environment, a stable internet connection, a computer, or a tablet with a good webcam and microphone. It is reasonable to have a backup plan in case the technology fails, such as providing your phone number to the interviewer in advance [8, 19].

Our survey examined current perceptions in residents about which specialties might be best suited for virtual-interviews, with the option to choose multiple answers. Over a third of residents in our survey thought that Physical Medicine & Rehabilitation, Psychiatry, Pediatrics, Radiology, Internal Medicine, and Family Medicine were specialties most conducive to virtual-interviews. The reason for this is not immediately clear. Notably, they are mainly non-surgical specialties. One valuable recommendation that was made by the participants in our study was that virtual-interviews could prove particularly beneficial for preliminary internship positions since their time commitment to the program is much less. A preliminary internship is a position offering only one year of training generally before entry into advanced specialty programs.

Participants of this study proposed that virtual-interviews are a worthy option in the screening process of residency application reviews. Although it appears current perceptions are not completely on board with the use of virtual-interviews in lieu of the customary in-person residency interviews, virtual-interviews may have other possible applications. For example, Bird et al. investigated using the AAMC standardized virtual interview to score a resident applicant's interpersonal skills and communication. They found that it provided unique information than what is currently available in academic metrics and can be used as part of a holistic screening process to find suitable applicants [20]. Perhaps instead of replacing in-person interviews, virtual-interviews can be applied as an additional screening tool to gauge important qualities such as personability and communication. However, news that was posted in November 2019 by AMA reported that a pilot program of Standardized Video Interview (SVI) in emergency medicine was not successful. The emergency medicine residents and program directors wrote a letter and emphasized their opinion. They stated that:” After three years of piloting the SVI, reviewing the data, and hearing from the members of our community, we respectfully oppose further study or use of the SVI.” So, AAMC decided not to offer the SVI for the ERAS 2021 application cycle [21].

Our study information could potentially be helpful for the residency program directors to see if virtual-interviews are a possibility for their applicants. The authors of this article currently have experience of using the virtual-interviews for the screening of the applicants. This has helped us save time, especially during the busy clinical practice for both parties. Moreover, we have been able to exclude the applicants who are not suitable for our program during a relatively short period. However, we have noticed that a high-speed internet connection and the available virtual platform for both parties are essential. The possibility of viewing the other party provides a greater sense of closeness and is the major advantage of video conference compared to a simple phone call [22]. However, the internet presents a certain degree of unpredictability since its network conditions may vary as a consequence of different factors [23]. Traffic fluctuations and network congestion may change end-to-end delay or may cause delay variation, bandwidth reduction, or packet loss bursts, which directly affects the quality of service of the running communications and can cause significant limitations [24].

Regardless of students' and residents' perceptions about virtual-interviews for residency application and despite their more interest in traditional in-person interviews, some circumstances leave us no choice between these two options. Since December 2019 when the novel coronavirus disease (COVID-19) outbreak was first described in Wuhan, China, many of the routine processes have been changed in the world [25]. As the social distance and travel limitation became a health advice to control the pandemic, several academic processes have also been impacted, including the residency interviews. Thus, despite AAMC news in November 2019, that reported residency programs will not continue with SVI, when the COVID-19 pandemic arrived, they had no choice but to shift toward the virtual-interviews in May 2020. AAMC strongly encouraged the medical school and residency program directors to conduct all residency interviews in a virtual setting, either by phone or through videoconferencing [1, 21].

### Future directions

The time period of this study was before the COVID-19 circumstances. However, in the existing condition, due to the COVID-19 pandemic, which travels are limited, and the doubt of whether large in-person meetings will be acceptable, educational programs should adapt and consider virtual interviews to facilitate this process for candidates [26–28]. The improvement in the United States residency selection process warrants preparation and guidelines for the residency programs to make this process smooth and optimum. Making structured interviews, protocols, and guidelines to improve the residency interview process will be more successful if the administrators consider the residency applicants' opinion. The authors believe that our study findings can help residency programs and AAMC, to include the applicants' current perceptions when preparing the national residency interview resources and guides. Knowing the residency applicants' attitude toward virtual-interviews is essential when preparing the programs' virtual-interview guides, especially when a national or international difficulty leaves us no choice but to use a virtual path to walk through our future.

### Study limitations

Our study had several limitations. Our sample size, the number of invited medical students and residents to respond to the questionnaire, was small, and it did not represent the whole residency applicants in the United States. The survey's response rate was another limitation of this study since the exact number of residents, and medical students who received this questionnaire was unclear. The response rate was calculated based on the reported count of residents and medical students in the programs who received the study's link and, therefore, it was not the exact response rate. Our study had a relatively low response rate and a higher participation from the female medical students, and consequently, this was another limitation. The lower rate of response was multifactorial. One reason was that we sent the survey to students in all four years of medical school; however, usually, only the students during the last year are concerned and participate in residency application activities. Another reason could be that the institutions didn't send the survey link to their students and residents, as they had the freedom to do so. Lastly, our study was not funded, and the survey had no reward, which can influence participation motivation. These limitations may impact our survey conclusion since our sample didn't represent all national and international residency applicants.

## Conclusions

Our study demonstrated that both medical students and residents have a similar perception about virtual-interviews, and both favor in-person interviews in normal circumstances. However, both groups agree that programs should offer the option of having virtual-interviews as an available choice. Distance to an interview location and the type of medical school were the factors that had a significant impact on perception of using virtual-interviews. Knowing the medical students’ attitude toward residency interviews and the national circumstances are essential when preparing the interview guides. Further larger scale studies are warranted to better understand the perception of residency applicants toward virtual-interviews to improve the residency interview process in the United States.
